# Network Diffusion-Based Prioritization of Autism Risk Genes Identifies Significantly Connected Gene Modules

**DOI:** 10.3389/fgene.2017.00129

**Published:** 2017-09-25

**Authors:** Ettore Mosca, Matteo Bersanelli, Matteo Gnocchi, Marco Moscatelli, Gastone Castellani, Luciano Milanesi, Alessandra Mezzelani

**Affiliations:** ^1^Bioinformatics Group, Institute of Biomedical Technologies, National Research Council of Italy, Segrate, Italy; ^2^Applied Physics Group, Department of Physics and Astronomy, University of Bologna, Bologna, Italy

**Keywords:** autism spectrum disorder, biological networks, network diffusion, data integration, gene module

## Abstract

Autism spectrum disorder (ASD) is marked by a strong genetic heterogeneity, which is underlined by the low overlap between ASD risk gene lists proposed in different studies. In this context, molecular networks can be used to analyze the results of several genome-wide studies in order to underline those network regions harboring genetic variations associated with ASD, the so-called “disease modules.” In this work, we used a recent network diffusion-based approach to jointly analyze multiple ASD risk gene lists. We defined genome-scale prioritizations of human genes in relation to ASD genes from multiple studies, found significantly connected gene modules associated with ASD and predicted genes functionally related to ASD risk genes. Most of them play a role in synapsis and neuronal development and function; many are related to syndromes that can be in comorbidity with ASD and the remaining are involved in epigenetics, cell cycle, cell adhesion and cancer.

## Introduction

“Autism spectrum disorder” (ASD) includes clinically and etiologically wide range of neurodevelopmental disorders such as the less severe disorders Asperger's syndrome and pervasive developmental disorder, not otherwise specified, as well as the most severe childhood disintegrative disorder. ASD symptoms are recognized mainly by the complex behavioral phenotype that manifests within the first 3 years of life: difficult in communication and social interaction, limited interests and repetitive behaviors (National Institute of Mental Health, 2013).

Genetics play a crucial role in autism pathogenesis (Devlin and Scherer, 2012). Indeed, ASD has a high-heritability index (0.85–0.92) (Monaco and Bailey, 2001), a significant sib recurrence risks (8.6%) and 64% concordance among monozygotic twins (Smalley, 1998). Thousands of causative or predisposing genetic variations have been found in ~30% of autistic patients (O'Roak et al., 2012), thus making autism a complex multifactorial disorder involving many genes and loci contributing to the phenotype. Genetic variations involved in ASD are chromosomal abnormalities (~5%), copy number variations (CNVs) (10–20%) and single-gene mutations (~5%) (Miles, 2011). Although the role of genetics in ASD etiology is recognized for ~70% of cases, the causative factor is still unknown.

The approaches currently used to disentangle the genetic complexity of ASDs include large genome-wide association studies (GWAS), CNV testing and genome sequencing. Interestingly, the application of these different approaches yielded many non-overlapping genes, which may suggest different molecular mechanisms within connected pathways (Pinto et al., 2014). The analysis of molecular interactions and pathways is therefore crucial for the interpretation of the results emerging from genome-scale studies on a pathology marked by a significant genetic heterogeneity. Indeed biological pathways associated with a specific pathology are likely to be more conserved than individual genetic variations, because multiple combinations of variations might perturb each pathway (Barabási et al., 2011). Network-based and pathway-based analyses can therefore provide a functional explanation to non-overlapping genes and narrow the targets for therapeutic intervention (Devlin and Scherer, 2012).

One of the problems that network-based analyses can solve is indeed the identification of the so-called disease modules, i.e., network regions associated with a disease (Barabási et al., 2011). Recently, molecular interaction networks have been used in the analysis of ASD genetic data to define gene networks associated with ASD. The identification of a subnetwork with desired properties from a large biological network (like the one formed by all protein-protein interactions) poses many challenges and therefore several approaches have been proposed (Mitra et al., 2013).

Regarding the integration of networks and ASD genetic data, Cristino et al. (2014) studied the interacting partners of genes known to be associated with ASDs and other related disorders; Noh et al. (2013) identified a significantly interconnected network of genes affected by CNVs; Li et al. (2014) studied the association between ASDs and genes forming topological communities (clusters of genes with a high density of connection between genes of the community and less connections with genes outside the community); Gilman et al. (2011) found functionally connected clusters of genes affected by CNVs.

Recently, network smoothing index (NSI) was proposed as a network-based quantity that allows to define a network region enriched with *a priori* information (Bersanelli et al., 2016). The NSI is based on network diffusion, a method that simulates the flow of a fluid throughout a network. NSI quantifies the network relevance of each gene in relation to a set of input genes (e.g., ASD genes), considering the whole network and mitigating the importance of hubs.

In this work, we use network diffusion and the NSI to propose a possible disease module for ASD, encompassing the network regions most frequently hit by molecular variations reported in several studies and collected in curated public databases. Moreover, our study introduces a network-based genome-wide prioritization of genes in relation to their known and predicted relevance for ASDs.

## Materials and methods

### Molecular interactions

STRING interactions were collected from STRING (version 10), a database of direct and indirect PPIs (Szklarczyk et al., 2015). Native identifiers were mapped to Entrez Gene (Brown et al., 2015) identifiers. In case multiple proteins mapped to the same gene identifier, only the pair of gene identifiers with the highest STRING confidence score was considered. A total of 11,535 genes and 207,157 links with confidence score ≥700 was retained.

### ASD risk genes

ASD risk genes were collected from The Simons Foundation Autism Research Initiative SFARI Gene database (Abrahams et al., 2013, version available in July 2015) and from Li et al. (2014).

SFARI Gene provides a publicly available database where genes are scored according to the strength of the evidence of gene's association with autism. In particular, genes are assigned to 7 categories (Supplementary Table 1): “syndromic” (S), “high confidence” (1), “strong candidate” (2), “suggestive evidence” (3), “Minimal evidence” (4), “Hypothesized” (5), and “Not supported” (6). SFARI genes were divided into to broad classes of high and low strength of association with ASD. Genes belonging to categories S, 1, 2, 3, 1S, 2S, 3S, 4S were included in SFARIh list, while genes of categories 4 and 5 were grouped into SFARIl. Native gene identifiers were converted to Entrez Gene (Brown et al., 2015) identifiers.

In addition to SFARIh genes, we considered 5 sources (namely dnCNVn, dnCNV3s, rCNV, dMUT, mMUT) of genes harboring CNVs and mutations associated with ASDs, proposed by large recent studies (Li et al., 2014). dnCNVn contains genes from an ASD-associated network composed of genes with *de novo* CNVs identified in 181 individuals and genes previously implicated in ASDs (Noh et al., 2013). dnCNV3s contains genes with *de novo* CNVs found in all three independent studies on more than 1,000 families (Levy et al., 2011; Sanders et al., 2011; Pinto et al., 2014). rCNV contains genes with rare CNVs found in a study involving approximately 1000 ASD individuals of European ancestry and matched controls (Pinto et al., 2010). dMUT and mMUT include genes with, respectively, disruptive and missense mutations (Neale et al., 2012; O'Roak et al., 2012; Sanders et al., 2012; Li et al., 2014).

Only genes occurring in STRING network were considered in network-based analyses (Table 1). Therefore, whenever appropriate, we will specifically refer to the original gene lists and the corresponding derived lists with only genes occurring in STRING network using suffixes “i” and “n0” respectively (e.g., SFARIh_i_, SFARIh_n0_).

**Table 1:**
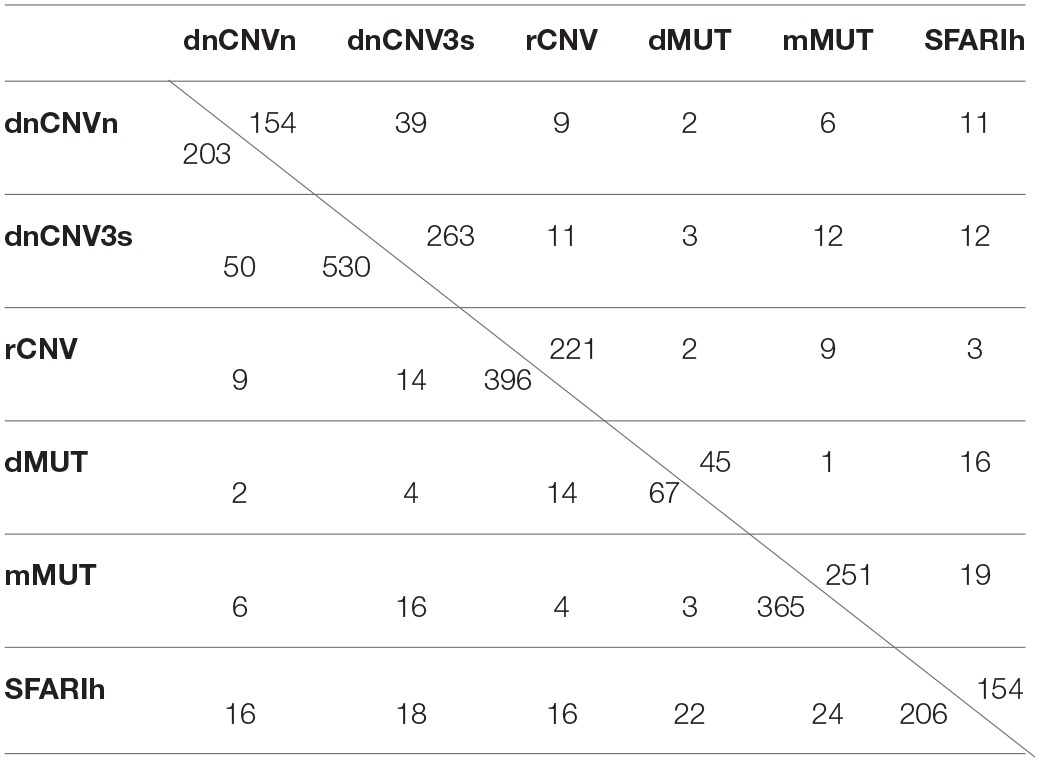
Overlap between lists of genes harboring variations associated with ASDs.

*Gene list size (diagonal) and number of genes co-occurring between pairs of lists (off-diagonal elements); lower triangle and diagonal (bottom): original gene lists; upper triangle and diagonal (top): original gene lists with only genes occurring in STRING network*.

### Network-based analysis

Given an input gene list *L* and a gene network encoded as the *n*-by-*n* symmetrically normalized adjacency matrix *W* (Bersanelli et al., 2016), the *n*-sized vector *X*_0_ was defined in order to have positive quantities only in its elements representing the genes in *L*, and null values for all the other genes. Network diffusion finds the vector *X*_∗_, in which the quantities initially available in *X*_0_ are subject to smoothing according to the pattern of interactions *W*. The vector *X*_∗_ was calculated using an iterative procedure (Zhou et al., 2004), as described in Bersanelli et al. (2016):

$$\begin{array}{l} X_{t+1}=\alpha W\;X_{t}+\left(1-\alpha \right)X_{0},\;X_{*}=\underset{t\to \infty }{\lim}X_{t}, \end{array}$$

where α [here set to 0.7 as in previous works (Bersanelli et al., 2016)] is a parameter that weights to which extent the initial information is retained or spread throughout the network. In the independent smoothing of each of the six ASD gene lists described above, genes belonging to the list were set to 1. In the joint analysis of all gene lists, genes belonging to SFARIh_n0_ were set to 1, while genes belonging to other lists were set to 0.5: this setting was chosen so that genes strongly associated with ASD had a higher priority.

For each gene *g*, the network smoothing index *S* quantifies the network proximity of *g* to genes marked by a positive value in *X*_0_, i.e., associated with ASD, as ratio between gene values after and before network diffusion:

$$\begin{array}{l} S\left(g\right)=\frac{X_{{}^{*}}\left(g\right)}{X_{0}\left(g\right)+\varepsilon } \end{array}$$

where ε is a small positive quantity that weights the importance of the initial values *X*_0_. In order to mitigate the tendency of hub genes to gather excessive amounts of information only because of their central position, the permutation-adjusted network smoothing index *S*_*p*_ was introduced as,

$$\begin{array}{l} S_{p}\left(g\right)=-log_{10}\left(p_{S}\left(g\right)\right)\cdot S\left(g\right) \end{array}$$

where *p*_*S*_(*g*) is an empirical *p*-value, computed using *K* permutations of *X*_0_, each one denoted as $X_{0}^{k}$, and the corresponding *S*^*k*^(*g*) (calculated using $X_{0}^{k}$):

$$\begin{array}{l} p_{S}\left(g\right)=\frac{1+\#\left\{S^{k}\left(g\right)\geq S\left(g\right)\right\}}{K+1}. \end{array}$$

In the analysis of the six ASD risk gene lists, ε values were defined in order to predict genes in network proximity to the input genes. Given a gene set of size *N*, ε was set in order to obtain, among the first *N* top ranking genes by *S*_*p*_, a ratio of 1:1 between (i) the number of input genes and (ii) the number of genes in network proximity to input genes. The resulting values were 0.21 for dnCNVn_n0_ and 0.19 for dnCNV3s_n0_, RcnV_n0_, dMUT_n0_, mMUT_n0_ and SFARIh_n0_. In the joint analysis of all gene lists ε was set equal to 1, because the analysis was mainly aimed at defining a network-based prioritization of the 956 input genes, rather than at predicting other genes in network proximity. In all these analyses we used *K* = 999.

Network resampling (NR) shows to which extent a network score, resulting from the combination of gene scores, is expected if links among genes are shuffled. Also in this case permutations are used to define the null model. Given a number *m* of genes at the top of a ranked gene list, NR consists of two steps. First, a non-decreasing quadratic objective function Ω(*m*) is defined:

$$\begin{array}{l} \Omega \left(m\right)=S_{p}^{T}\left(m\right)\cdot A_{m}\cdot S_{p}\left(m\right), \end{array}$$

where *S*_*p*_(*m*) is the vector referring to the first *m*-scoring genes and *A*_*m*_ is the adjacency matrix between such genes. In the second step, *q* permutations of *A*_*m*_ are defined keeping the same degree distribution. Lastly, an empirical *p*-value (*p*_*N*_) is calculated to quantify the fraction of times the objective function calculated on a permuted network, Ω^k^(*m*), is greater than or equal to Ω(*m*). The procedure is repeated for different *m*, providing an overview on whether gene links and gene scores determine significant network scores when moving down in the ranked gene list (see figures below).

Network resampling (NR) was applied to genes ranked by *S*_*p*_ in descending order and using a total of 200 permutations, which was enough to underline the presence of significantly connected components (gene modules).

### Pathway analysis

Pathway analysis was carried out using over-representation analysis (ORA). ORA estimates the significance of a pathway in relation to an input gene list, calculating the hypergeometric probability of finding the observed number of input genes that are also members of the considered pathway, in the context of a background set of genes. As a background we considered all the genes occurring in original lists and all genes occurring in the gene network. Gene-pathway associations were downloaded from NCBI Biosystems (version: February 2017) (Geer et al., 2010); in particular, only pathways (gene sets) with a number of genes between 10 and 200 were considered. Hypergeometric probabilities were calculated using “phyper” and “dhyper” R functions, and were corrected for multiple hypotheses testing using the Benjamini-Hochberg method, implemented in “p.adjust” R routine. The similarity between two gene sets (*A, B*) was calculated using the overlap coefficient: *o* = |*A* ∩ *B*|/ min(|*A*|, |*B*|).

## Results

### Network location of genes associated with ASDs in SFARI database

With the aim of characterizing the functional relations among SFARI genes and predict relevant risk genes for ASDs, we considered direct and indirect protein-protein interactions (PPI) and quantified, *via* the permutation-adjusted NSI (*Sp*) (Bersanelli et al., 2016), the network proximity of each human gene in relation to the network location of 154 genes reported as strongly associated with ASD (SFARIh_n0_ list, Table 1).

**Figure 1:**
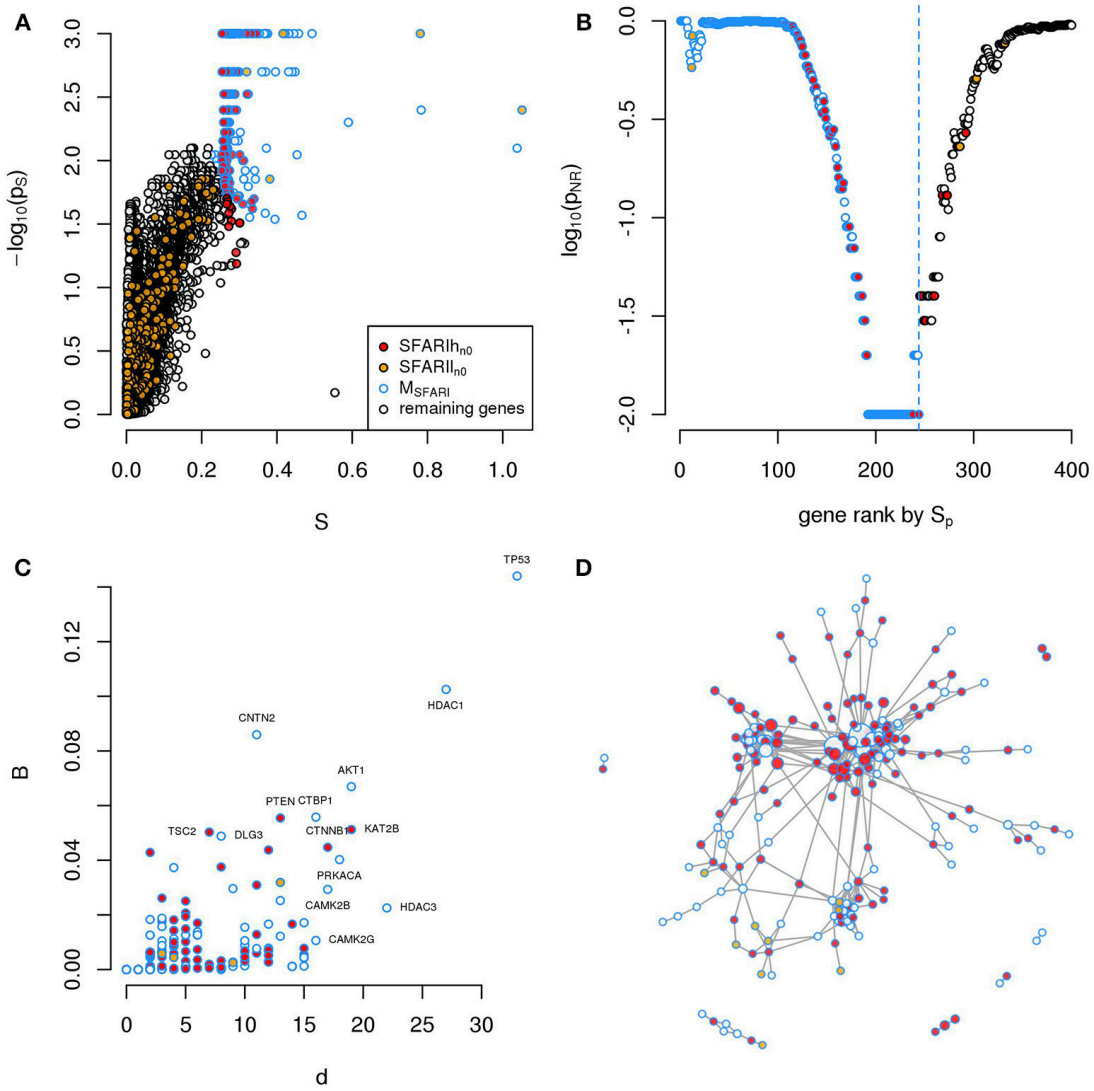
A significantly connected gene module based on SFARIh genes. The network-diffusion based analysis of SFARIh genes (red) leads to the definition of a significantly connected gene module of 244 genes (blue border). **(A)** Network smoothing index and corresponding estimated *p*-value (*p*_*s*_). **(B)** For each rank *n* (horizontal axis), the estimated *p*-value (*p*_*NR*_) that quantifies the significance of the gene network defined by the genes ranked up to the *n*-th rank. **(C)** Number of interactions (*d*) and normalized betweenness (*B*) of genes in M_SFARI_. **(D)** Visualization of M_SFARI_ gene module, in which genes are circles and PPI are links; circle size is proportional to gene degree. **(A–D)** *G*: all genes included in the STRING network.

We found several genes in significant network proximity to SFARIh_n0_ genes, with high *S* and low *p*_*S*_ (Figure 1A and Supplementary Table 2). Interestingly, among these genes, we found a significant number of genes having minimal/hypothetical evidences of association with autism in SFARI (SFARIl_n0_) (Table 2).

**Table 2:** Number of SFARIl genes in network proximity to SFARIh genes.

**|M ∩ SFARIl|**	**|M|**	**|G-M|**	**|SFARIl|**	**E(|M ∩ SFARIl|)**	***p***
2	10	11,371	216	0.190	1.46·10^−2^
9	100	11,281	216	1.90	1.15·10^−4^
9^*^	102^*^	11,279^*^	216^*^	1.94^*^	1.34·10^−4^^*^
20	300	11,081	216	5.69	1.07·10^−6^
22	400	10,981	216	7.59	7.27·10^−6^
26	500	10,881	216	9.49	2.87·10^−6^

*M, genes in network proximity to SFARIh genes; G, all genes; |M ∩ SFARIl| size of the intersection between M and SFARIl; $E\left(\left|M\;\cap \;SFARIl\right|\right)=\frac{\left|M\right|}{\left|G\right|}\cdot |SFARIl|$, expected |M ∩ SFARIl|; p: probability that |M ∩ SFARIl| is greater than or equal to the observed value in a hypergeometric experiment; the asterisks underline the overlap obtained using the top ranking 244 genes ranked by S_p_ (102 after removing SFARIh genes) composing a significantly connected gene module (M_SFARI_) (Figure 1)*.

In order to assess whether genes ranked by *S*_*p*_ formed a significantly connected gene module, we applied the NR approach (Bersanelli et al., 2016). We observed a significantly connected gene module (M_SFARI_) resulting from the top 244 genes (Figures 1B–D). This module includes 142 (out of 154) SFARIh_n0_ genes, 9 SFARIl_n0_ genes and 93 genes not in SFARI.

These 93 genes include regulators of synaptic development and plasticity, are involved in syndromic conditions in comorbidity with ASD, regulate epigenetic mechanisms and a few are associated to cancer (Supplementary Table 2). For example, among the 93 genes that have a relevant position within the module (Figure 1C) we found cancer genes that control cell proliferation, [e.g., Tumor Protein P53 (TP53), AKT Serine/Threonine Kinase 1 (AKT1), Mechanistic Target Of Rapamycin (MTOR), C-Terminal Binding Protein 1 (CTBP1)], a process that was recently proposed as a common denominator of cancer and ASDs (Crawley et al., 2016), and genes with relevant role for brain function e.g., histone deacetylase-1 (HDAC1), histone deacetylase-3 HDAC3 (Volmar and Wahlestedt, 2015) and contactin-2 (CNTN2) (Anderson et al., 2012). These 93 predicted genes act as a bridge between SFARIh genes that were not directly linked in STRING (Figure 1D).

Interestingly, while this manuscript was under review, 3 out of the 93 predicted genes were added to the SFARI database independently from our study. Namely, HADAC3 was added among “hypothesized” genes, while TANC2 and PPP2R1B among “minimal evidence” genes.

### Genes in network proximity to genes harboring variations associated with ASD

**Figure 2:**
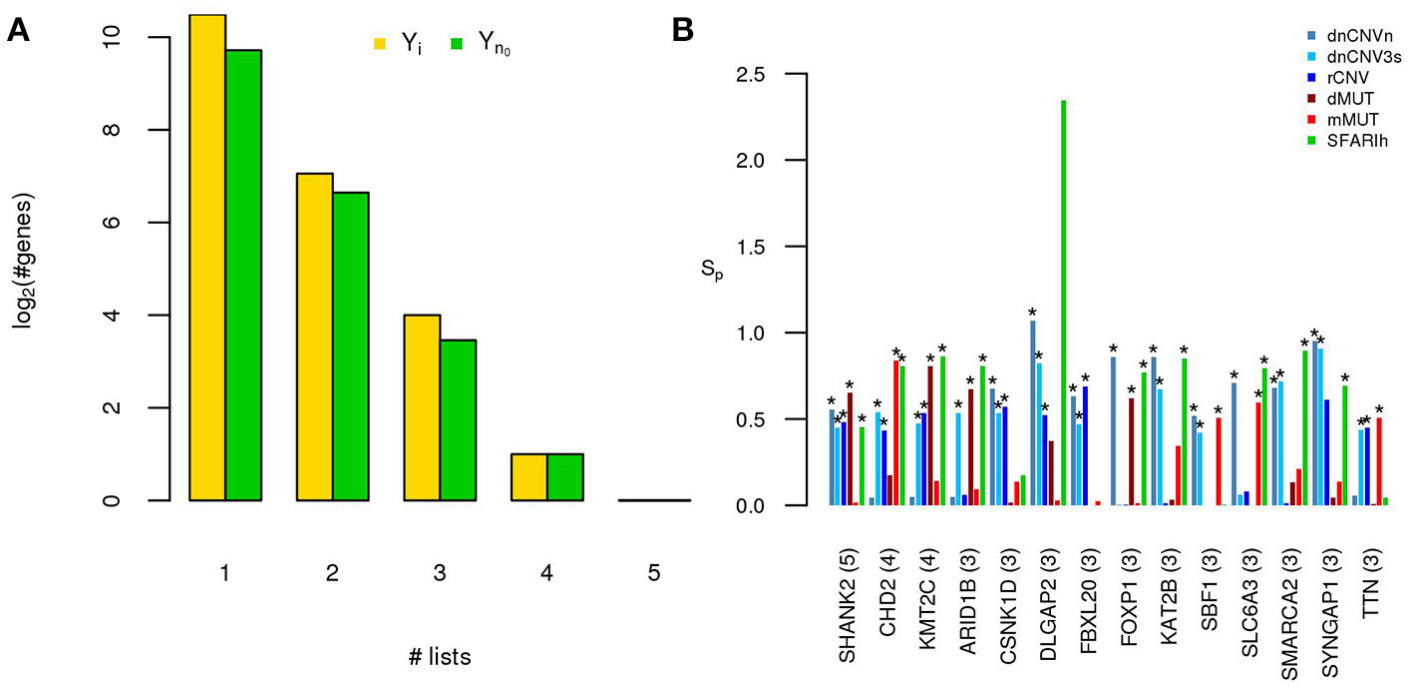
A small number of ASD risk genes is found in more than one large study. **(A)** Number of risk genes found in 1 or more of the six gene lists on ASD; *i*: original gene lists; *n0*: original gene lists wit only genes occurring in the STRING network. **(B)** Permutation-adjusted network smoothing index of the 14 genes occurring in 3 or more original lists (the number is reported between parenthesis); the asterisk (^*^) indicates genes of the corresponding original list.

In addition to the SFARIh gene list, we considered five other lists of genes found altered in ASD subjects by previous studies. These lists vary in size from 67 to 530 and from 45 to 263 genes after integration with STRING gene network. The percent overlap between lists is low (Table 1) and most of the genes occur only in one list (Figure 2). In this situation, as introduced earlier, the information on how gene products interact to regulate biological functions can be used to explain the heterogeneity of ASD risk gene lists. Firstly, we analyzed each list separately to underline the specificities and commonalities of each list. Subsequently, we used network information to define a prioritization among all ASD risk genes proposed in the considered studies (union of all gene lists).

**Figure 3:**
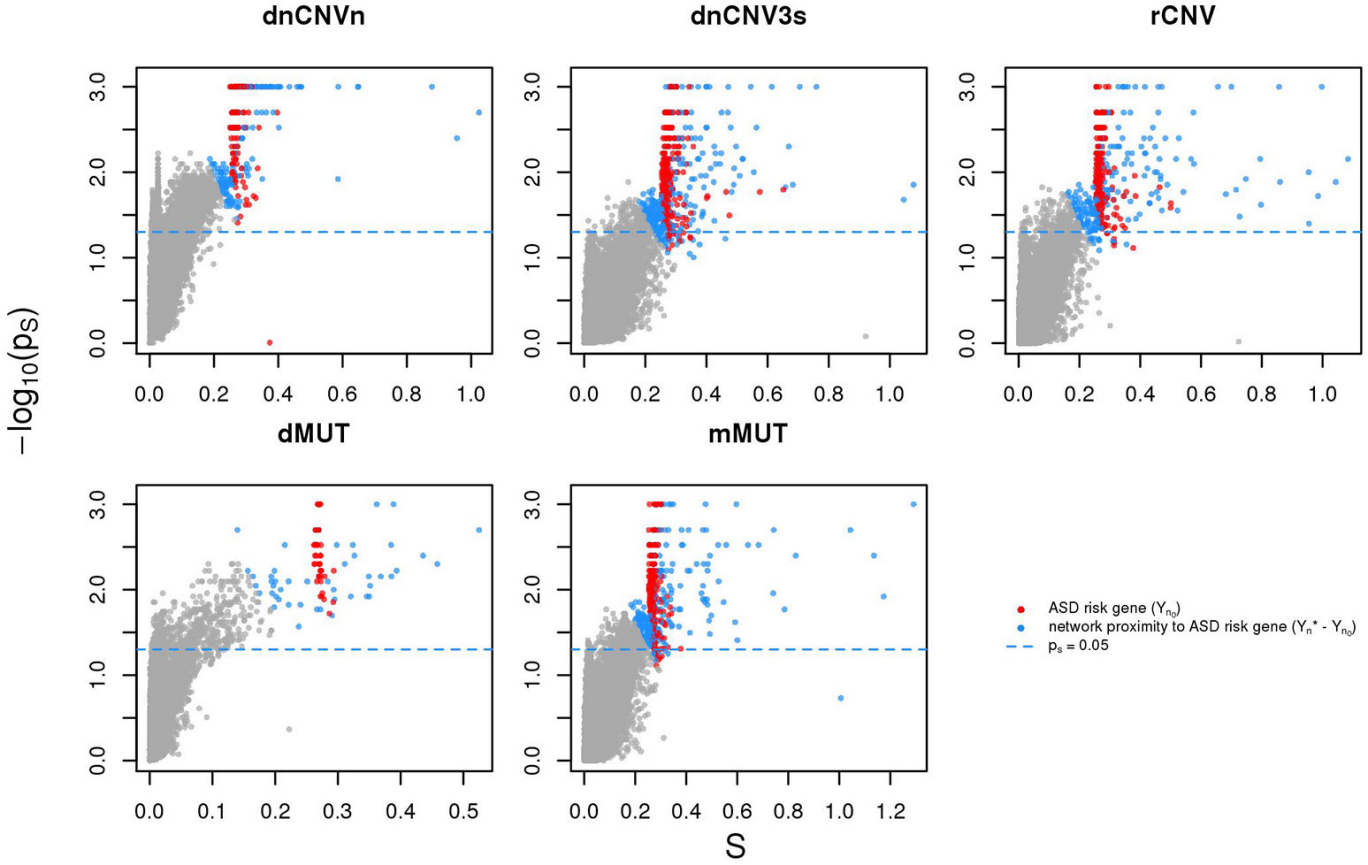
Genes in network proximity to ASD risk genes. The network smoothing index (*S*) and the corresponding *p* value (*p*_*s*_) are the two factors that make up *Sp*. Blue: genes belonging to the corresponding original list (sources); red: genes in network proximity to sources; gray: remaining genes.

We calculated the *Sp* of all genes in STRING network considering as input each of the six ASD gene lists (SFARIh_n0_, dnCNVn_n0_, dnCNV3s_n0_, rCNV_n0_, dMUT_n0_, and mMUT_n0_) and selected the top 2*n* genes ranked by decreasing values of *Sp*, where *n* is list size (Supplementary Table 3). Note that almost all these genes are in significant network proximity (*p*_*S*_ < 0.05) to the corresponding input genes (Figure 3 and Supplementary Table 3), which are also ranked among the top 2*n* genes. For convenience we will refer to these network-based gene lists—which contains input and predicted genes—as ${\text{SFARIh}}_{{\text{n}}^{*}}$, ${\text{dnCNVn}}_{{\text{n}}^{*}}$, dnCNV3${\text{dnCNV3s}}_{{\text{n}}^{*}}$, ${\text{rCNV}}_{{\text{n}}^{*}}$, ${\text{dMUT}}_{{\text{n}}^{*}}$, and ${\text{mMUT}}_{{\text{n}}^{*}}$.

Only 14 genes occur in three or more input gene lists (Figure 2). Among those genes, at least DLGAP2 (Discs Large Homolog Associated Protein 2) and SYNGAP1 (Synaptic Ras GTPase Activating Protein 1) are worth mentioning. In fact, DLGAP2, a post-synaptic density protein with probable implication in ASD pathogenesis (Chien et al., 2013) scored as “minimal evidence” in SFARI (SFARIl), is part of all three CNV lists and was predicted as functionally related to SFARIh genes and dMUT genes. Furthermore, DLGAP1 (Discs Large Homolog Associated Protein-1) was predicted as functionally related to genes of 3 input lists, including SFARIh (Figure 2B). Similarly, SYNGAP1, which codes an autism related brain-specific synaptic Ras GTP-activating protein (Berryer et al., 2013), occur in three input lists (dnCNVhc, dnCNV3s and SFARIh) and was predicted as functionally related to genes harboring rCNVs.

Globally, a total of 913 genes were predicted as functionally related to at least one ASD risk gene list (Table 3 and Supplementary Table 4). Interestingly, 106 of these genes were already proposed as ASD risk genes in 1 or more studies: for example, CTNNB1 (Catenin-Beta1) encoding a protein part of the adherens junctions complex, NRXN1 (Neurexin1), NLGN4X (Neuroligin4X), encoding a pre-synaptic and post-synaptic protein, respectively, and the tumor suppressor PTEN (Phosphatase And Tensin Homolog), were predicted as functionally related to 2 gene lists and included as risk genes in other 2 lists.

**Table 3:** Co-occurrence of genes in network proximity to ASD risk genes from one or more sources.

		**Input (*n*_*i*_)**				
		**0**	**1**	**2**	**3**	**#**
**Predicted (** *n*_*p*_ **)**	**1**	691	68	14	2	775
	**2**	89	15	4	1	109
	**3**	18	2	0	0	20
	**4**	6	0	0	0	6
	**5**	3	0	0	0	3
	**#**	807	85	18	3	913

*A total of 913 genes were predicted to be in network proximity to ASD risk genes of one or more studies (rows) where could appear as ASD risk genes (columns). For example, 15 genes were predicted in network proximity to ASD risk genes of 2 studies and were proposed as ASD risk genes in 1 study (n_i_ = 1, n_p_ = 2); #: row or column sum*.

**Figure 4:**
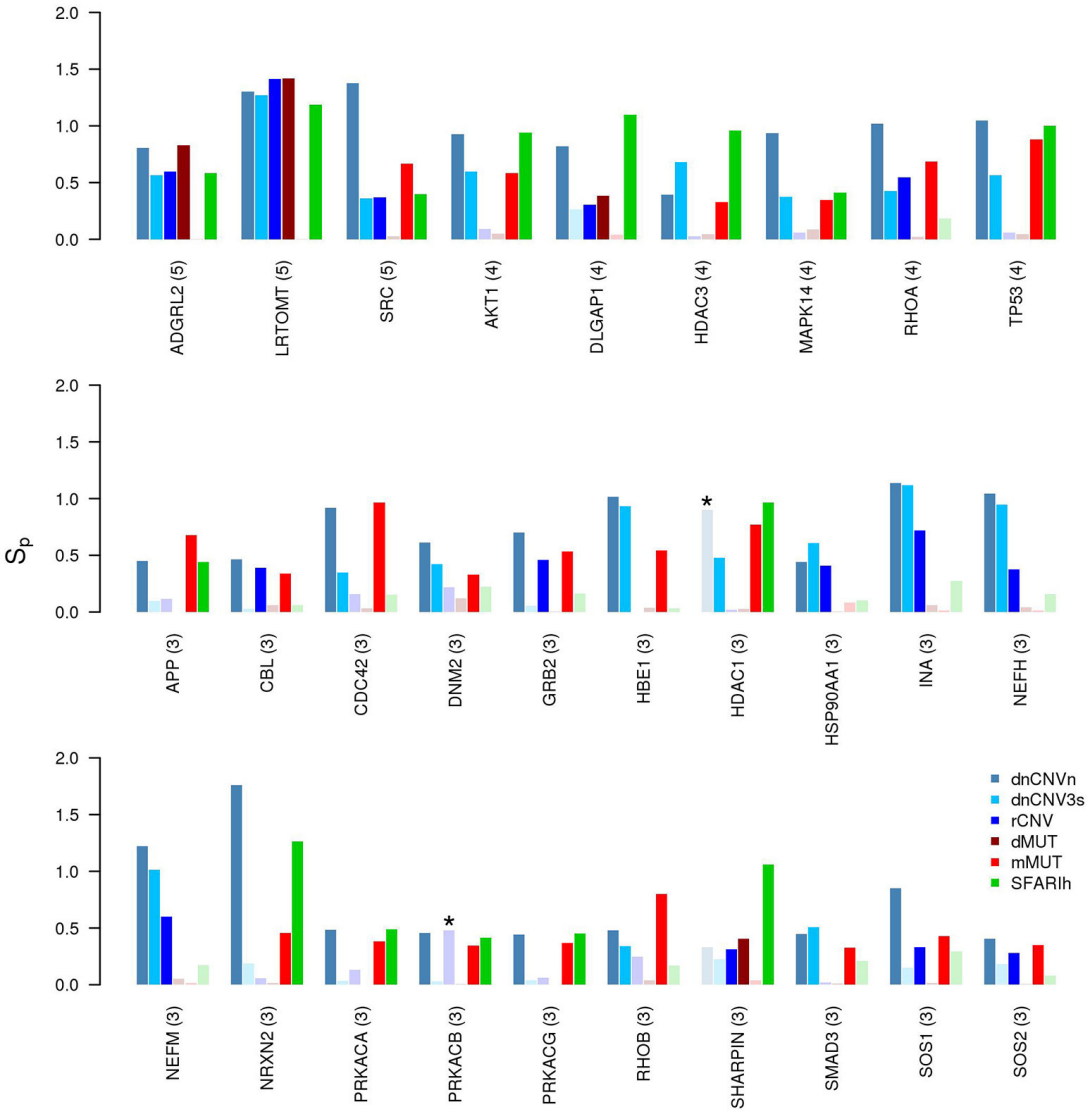
Genes in network proximity to ASD risk genes from 3 or more studies. Permutation-adjusted network smoothing index (*Sp*) of the 29 genes predicted as functionally related to 3 or more ASD risk gene lists (the number is reported between parenthesis); the asterisk (^*^) indicates genes of the corresponding original list; faded colors indicate input ASD risk genes or genes with no significant *Sp* values.

We have also found genes that were not included in any input gene list, but were predicted to be in relevant network proximity to multiple gene lists. For example, 27 were predicted as functionally related to three gene lists (Figure 4); among these, ADGRL2 (adhesion G protein-coupled receptor L2), LRTOM (leucine rich transmembrane and O-methyltransferase domain containing) and SRC (Proto-Oncogene Non-Receptor Tyrosine Kinase SRC) were predicted in functional relation to 5 lists.

Many of the 29 genes predicted as functionally related to three or more lists—27 genes not included in any input list and 2 included in one study—take part in many PPIs, implicating they are central in the PPI network (e.g., TP53, AKT1). The significance of their *Sp* suggests that these genes were not only selected in relation to their centrality, but also because their network distance to ASD risk genes is lower than expected by chance (Figure 3 and Supplementary Table 3). A further observation that supports this hypothesis is that these genes establish a number of interactions with ASD risk genes that is higher than expected (*p* < 0.05, hypergeometric test) (Table 4). From a network point of view, these 29 genes are “surrounded” by 369 ASD risk genes (first order neighbors).

**Table 4:** Hub genes predicted in network proximity to ASD risk genes of three or more studies establish a significant number of interactions with ASD risk genes.

**Gene and function**	**Symbol**	**Band**	**# Studies**	***n_*p*_***	**|*I*|**	**|A ∩ I|**	***p***
*SRC Proto-Oncogene, Non-Receptor Tyrosine Kinase* Nonreceptor tyrosine kinase, frequently implicated in cancer	SRC	20q11.23	0	5	532	82	2.00·10^−8^
*Tumor Protein P53* Involved in cell cycle regulation where negatively regulate cell division. Mutations in this gene are associated with a variety of human cancers	TP53	17p13.1	25	4	719	92	1.36·10^−5^
*AKT Serine/Threonine Kinase 1* Implicated in the regulation of cell growth, proliferation, survival and differentiation (OMIM 164730)	AKT1	14q32.33	25	4	589	73	2.90·10^−4^
*Ras Homolog Family Member A* Regulates remodeling of the actin cytoskeleton during cell morphogenesis and motility. Overexpression of this gene is associated with tumor cell proliferation and metastasis	RHOA	3p21.31	13	4	406	65	1.53·10^−7^
*Mitogen-Activated Protein Kinase 14* is a member of the MAP kinase family that are involved in cellular processes such as proliferation, differentiation, transcription regulation and development	MAPK14	6p21.31	11	4	333	44	1.30·10^−3^
*Histone Deacetylase 3* belongs to the histone deacetylase family and represses transcription when tethered to a promoter; down-regulates p53 function and thus modulate cell growth and apoptosis	HDAC3	5q31.3	8	4	275	43	3.54·10^−5^
*Heat Shock Protein 90 Alpha Family Class A Member 1* is a molecular chaperone involved in signal transduction, protein folding, protein degradation, and morphologic evolution.	HSP90AA1	14q32.31	10	3	589	65	9.99·10^−3^
*Amyloid Beta Precursor Protein* Is involved in promoting transcriptional activation; can participate in the formation of amyloid plaques of Alzheimer disease.	APP	21q21.3	15	3	363	42	1.68·10^−2^
*Histone Deacetylase 1* Is a histone deacetylase and represses transcription; interacts with retinoblastoma tumor-suppressor protein to control cell proliferation and differentiation; modulates p53 effect on cell growth and apoptosis.	HDAC1	1p35.2	12	3	351	48	3.69·10^−4^
*Cell Division Cycle 42* Acts in cell morphology, migration, endocytosis and cell cycle progression.	CDC42	1p36.12	9	3	336	56	2.97·10^−7^
*Growth Factor Receptor Bound Protein 2* is involved in the signal transduction pathway.	GRB2	17q25.1	21	3	279	42	1.06·10^−4^
*Protein Kinase CAMP-Activated Catalytic Subunit Alpha* phosphorylates proteins and substrates, changing their activity; contributes to the control glucose metabolism, cell division, and contextual memory; developmental changes in synapse morphology.	PRKACA	19p13.12	14	3	278	41	2.00·10^−4^
*SOS Ras/Rac Guanine Nucleotide Exchange Factor 1* participates in signal transduction pathways.	SOS1	2p22.1	14	3	260	50	1.26·10^−8^
*SMAD Family Member 3* signal transducer and transcriptional modulator that mediates multiple signaling pathways probably involved in carcinogenesis	SMAD3	15q22.33	1	3	241	33	2.83·10^−3^
*Protein Kinase CAMP-Activated Catalytic Subunit Beta* is a member of the serine/threonine protein kinase family involved in cell proliferaton and differentiation	PRKACB	1p31.1	22	3	213	28	9.81·10^−3^
*Cbl Proto-Oncogene* targets substrates for degradation by the proteasome; is mutated or translocated in many cancers	CBL	11q23.3	12	3	203	37	3.77·10^−6^
*Protein Kinase CAMP-Activated Catalytic Subunit Gamma* is involved in the regulation of lipid and glucose metabolism and in the memory formation signaling cascade	PRKACG	9q21.11	7	3	185	27	2.70·10^−3^
*Ras Homolog Family Member B* Involved in intracellular protein trafficking of a number of proteins; plays a negative role in tumorigenesis	RHOB	2p24.1	8	3	174	38	2.19·10^−8^
*SOS Ras/Rho Guanine Nucleotide Exchange Factor 2* is involved in the positive regulation of ras proteins	SOS2	14q21.3	12	3	109	24	7.36·10^−6^
*Dynamin 2* produces microtubule bundles and binds and hydrolyzes GTP; regulates neuron morphology, axon growth; vesicular trafficking processes and cytokinesis	DNM2	19p13.2	19	3	84	21	3.27·10^−6^

*Gene and function from GeneCards (Safran et al., 2003) and OMIM (Amberger et al., 2015); Band: cytogenetic band associated with genetic variations in SFARI (Abrahams et al., 2013); # Studies: number of studies supporting the genetic variations observed in the cytogenetic bands; I: interactors; A: ASD risk genes, |A| = 956; n_p_: number of ASD risk gene sets the gene is network proximity to; I: interactors; p: probability that |A ∩ I| is greater than or equal to the observed value in a hypergeometric experiment; the total number of genes composing the network is 11,535*.

**Figure 5:**
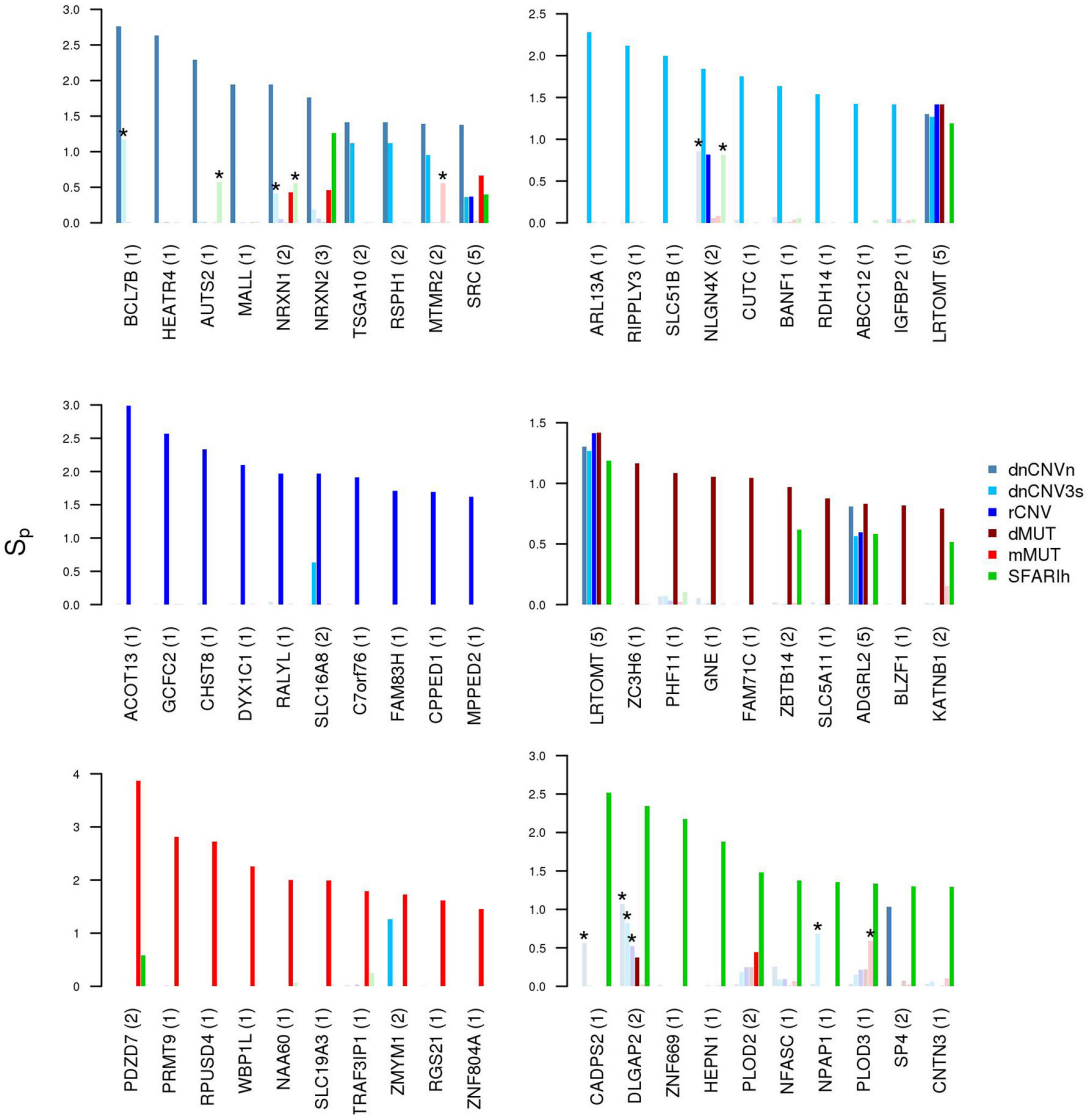
Genes in network proximity to ASD risk genes from each study. Top 10 genes with the highest permutation-adjusted network smoothing index (*Sp*) calculated in the analysis of each ASD risk gene list; the number of lists to which the gene was predicted as network proximal is reported between parenthesis; the asterisk (^*^) indicates genes of the corresponding original list; faded colors indicate input ASD risk genes or genes with no significant *Sp* values.

It is also worth mentioning that several genes resulting with the highest network proximity score to each list tend to be list specific (Figure 5).

**Figure 6:**
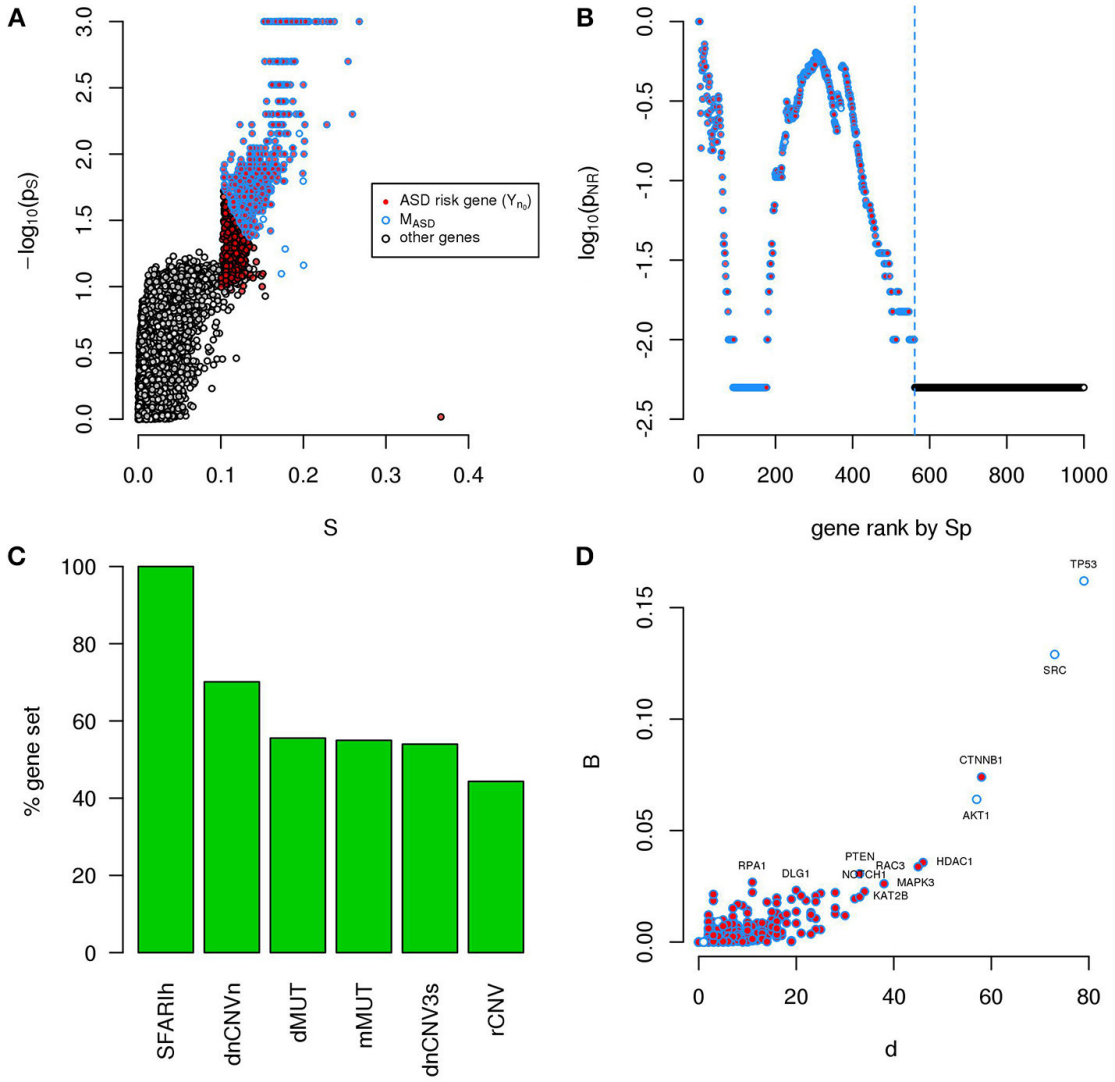
A significantly connected gene module based on ASD risk genes from 6 sources. The network-diffusion based analysis of 956 ASD risk genes from 6 sources (red) leads to the definition of a significantly connected gene module (M_ASD_) of 561 genes (blue border). **(A)** Network smoothing index (*S*) and corresponding *p*-value (*p*_*s*_). **(B)** For each rank *n* (horizontal axis), the estimated *p*-value (*p*_*NR*_) that quantifies the significance of the gene network defined by the genes ranked up to the *n*-th rank is reported on the vertical axis. **(C)** Percent of ASD genes of original lists included in M_ASD_. **(D)** Number of interactions (*d*) and betweenness (B) of genes in M_ASD_. **(A–D**) *G*: all genes included in the PPI network.

A total of 956 unique ASD risk genes occur in the 6 input lists. We calculated the *Sp* of all genes relative to these 956 genes (Figure 6A) and, by means of NR, found a significantly connected component of 561 genes (Figure 6B and Supplementary Table 5). This gene module (M_ASD_) includes all SFARIh genes, 70% of genes occurring in dnCNV_n list, approximately 50% of the other gene lists (Figure 6C), 26 genes in SFARIl and 8 genes that do not belong to the input list. Among these 8 genes, we find the already mentioned AKT1, TP53, and SRC, which occupy a central role in the PPI network and were also predicted during the independent analysis of each input list (Figures 4, 6D).

### Pathway analysis of gene lists associated with ASDs

**Figure 7:**
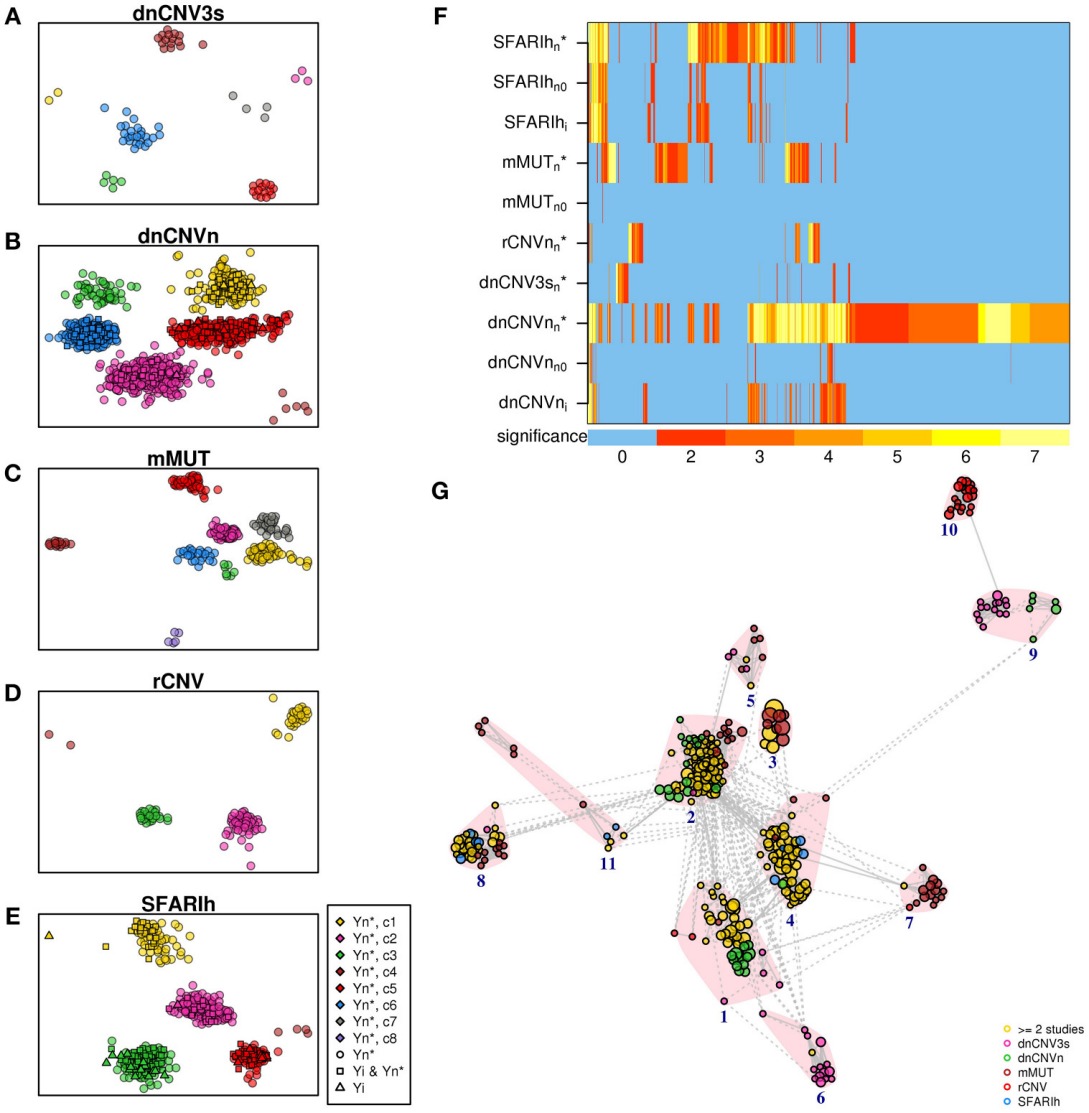
Significant pathways enriched in genes associated with ASD. **(A–E)** Pathways found in the analysis of each original list (*i*) and corresponding predicted genes (n^*^). **(F)** Heatmap of all the pathways found in the analysis of the lists reported on the rows; also pathways found analyzing the original lists without genes not occurring in the STRING network (*n*_0_) are represented; the color bar indicates the −log_10_(*p*) of the hypergeomtric *p*-value, adjusted for multiple hypothesis testing; the number 7 indicates *p* ≤ 10^−7^. **(G)** Enrichment map; vertex size is proportional to pathway significance (adjusted *p*-value); links are reported only for overlap coefficients >0.5; for each pathway, only the links with the top 5 most similar pathways are drawn. **(A–G)** See Supplementary Table 6.

We carried out an over-representation analysis to characterize original gene lists and network-based gene lists in terms of pathways. We observed significant pathways (at adjusted *p* < 0.01) in only two of the six original gene lists (SFARIh_i_ and dnCNVn_i_) and in five network-based lists (${\text{SFARIh}}_{{\text{n}}^{*}}$, ${\text{mMUT}}_{{\text{n}}^{*}}$, ${\text{rCNV}}_{{\text{n}}^{*}}$, ${\text{dnCNV3s}}_{{\text{n}}^{*}}$, and ${\text{dnCNVn}}_{{\text{n}}^{*}}$) (Supplementary Table 6). Overall, we obtained a much higher number of pathways in network-based lists than in original ones, despite the number of tested genes was similar between the former and the latter ones. Therefore, the observed enrichment in pathways can be mainly brought back to the network-based analysis, since, by definition, it prioritizes genes functionally related to those considered in input. Further, apart from a single exception in SFARIh, pathways found only in original lists were similar, in terms of gene content, to pathways found also in network-based lists (Figures 7A–F). In other words, network-based analysis resulted in an enrichment at pathway level with a very limited loss of information. Interestingly, pathways found in original gene lists, and lost due to genes for which network-based analysis was not applicable, were recovered by network-based analysis (compare rows labeled with suffix “i”, “n0”, and “n^*^” in Figure 7F).

We summarized the significant pathways found in each lists in a unique pathway network, the so-called enrichment map (Merico et al., 2010). Specifically, we took into account up to 20 of the most significant pathways as representatives of each pathway cluster found in each list. This selection resulted in a total of 366 pathways, clustered in 11 groups: transmission across synapses (clusters 1 and 4), signal transduction (2), Rho GTPase and apoptosis (3), inositol phosphate metabolism (5), ER-associated degradation process (6), ion transport (7), chromatin remodeling (8), oxygen transport (9), proteoglycan biosynthesis (10) and Wnt signaling (11). While the majority of pathways were found in more than 1 list (Figure 7G, yellow circles), some pathway clusters were composed of pathways uniquely associated with one list. For instance, proteoglycan biosynthesis was specifically associated with rCNV, ion transport with mMUT, oxygen transport with dnCNV3s and dnCNVn.

## Discussion

Recently, the knowledge of molecular interactions has been used for the interpretation of genetic data on ASDs. In comparison to previous works, we analyzed multiple ASD risk gene lists proposed in large studies, for a total of approximately 1,000 genes. We observed a low overlap between ASD risk gene lists. Whether this heterogeneity reflects the biology of ASD or is the result of confounding factors, the analysis of network proximity between genes underlines the ASD risk genes that are also in functional relation and lead to the identification of modules of functionally related genes hit by genetic variations. The main limitation of a network-based analysis such as ours is the availability of *a priori* annotations required for the definition of the genome-scale network. In this work we considered both direct (physical) and indirect (functional) high confidence PPI from STRING, which allowed us to analyze 11,535 human genes. Note that the use of direct and indirect STRING interactions showed good performances in prioritizing candidate disease genes (Köhler et al., 2008). Moreover, unlike other network-based works on ASD genetic data, we used network diffusion to quantify network proximity between ASD risk genes and other genes. Network diffusion (a global approach) considers the whole network topology in its full complexity and, therefore, has better performances than local approaches (e.g., direct neighborhood or shortest path length; Wang et al., 2011). Lastly, we underlined ASD risk gene modules without constraining the search to topological communities. In fact, there is no guarantee that topological communities are able to capture disease modules (Ghiassian et al., 2015). Hence, we quantified the significance of the observed network proximity scores in comparison to random networks of the same degree distribution (Bersanelli et al., 2016).

Our work provides a network-based prioritization of human genes associated with ASD by previous studies. We extracted a module of 244 genes in network proximity to genes reported in SFARI as strongly associated with ASD. Interestingly, the module contains a significant number of genes proposed as possibly involved in ASD (categorized as “minimal evidence” and “hypothesized” in SFARI) and another 93 genes not scored in SFARI (Supplementary Table 2). While this manuscript was under review, 3 of these 93 genes were included in SFARI independently from our study.

From the 93 genes, 16 genes are involved in synaptogenesis and synaptic plasticity or transmission, and alterations in structure and function of neuronal synapses are well known causes of ASD. Among these, APLP2 also regulates proper progression of neuronal differentiation program during cortical development (Shariati et al., 2013), is involved in Alzheimer disease and interacts with CNTN in neurodevelopment and diseases (Osterfield et al., 2008). Then again, the 3 genes, CACNA2D1, CACNB1, CACNG1 induce the repression of the downstream regulatory element antagonist modulator (DREAM) and the expression of the neuropeptide dynorphin (DYN). DREAM plays a role in synaptic plasticity and behavioral memory (Wu et al., 2010), while DYN is involved in behavioral symptoms characteristic of human depressive disorders (Knoll and Carlezon, 2010). Also CACNG3, a calcium channel protein, regulates the function of AMPA-selective glutamate receptors and mediates synaptic transmission in CNS while FLRT3 takes part in a trans-synaptic complex (Lu et al., 2015). Eight genes are involved in neuronal differentiation, neurodevelopment and neuronal function. More specifically, AKT1 is a downstream mediator of the PI3K pathway that regulates synaptic formation and plasticity and which imbalance leads to autism and schizophrenia (Enriquez-Barreto and Morales, 2016); genetic variations in contactins (CNTN) have been described in association with neurodevelopmental disorders, including autism. Specifically, CNTN1 and CNTN2 are members of the presynaptic *NRXN* superfamily and 13 rare non-synonymous variants of CNTN2 have been found in ASDs patients while mice with *Cntn5* mutations show an abnormal audiogenic response due to defects in the formation of synapse in auditory neurons (Cottrell et al., 2011; Chen et al., 2014).

Many of the 93 genes are involved in syndromic comorbidities, including auditory and visual senses deficit, epilepsy, mental retardation and psychiatric conditions that affect nearly three-quarters of children with ASD. For instance, CAMK2G and PDZD7 are involved in Usher Syndrome the most common condition leading to deafness and blindness, as well as DNMT1 has a role in DNMT1-Related Dementia, Deafness, and Sensory Neuropathy (Vernon and Rhodes, 2009) and LRTOMT in deafness. Again, MYL7 mutations are associated with Fechtner Syndrome which features include hearing loss and eye abnormalities. SP4 is involved in bipolar disorder and schizophrenia while ANK3, ACSL4, DLG3 are associated with mental retardation and, interestingly, recalling the high male prevalence of ASD, the latter two map on X-cromosome; NHS also maps on X-cromosome and mutations in this gene cause Nance-Horan Syndrome characterized by congenital cataract leading to vision loss; in males mild or moderate mental retardation may also occur and ASD have also been described in few patients (Toutain et al., 1997). Mutations in NAGLU and HGSNAT cause the Sanfilippo Syndrome (also called mucopolysaccharidosis Type III) often misdiagnosed with idiopathic developmental delay, attention deficit/hyperactivity disorder and/or ASD (Wijburg et al., 2013). QDPR mutations provoke hyperphenylalaninemia (Trujillano et al., 2014), (also called atypical phenylchetonuria (PKU), a genetic metabolic disease provoking postnatal cognitive deficit due to the neurotoxic effect of hyperphenylalaninemia; interestingly, PKU could be a comorbid condition of ASD, although with low prevalence (Baieli et al., 2003). MKRN3 is associated with Prader Willy Syndrome, NPAP1 both with Prader Willy Syndrome and Angelman Syndrome while DSCAML1 with Down Syndrome. These syndromes are characterized by mental retardation and can have co-occurring ASDs (Peters et al., 2004; Capone et al., 2005; Dykens et al., 2011). KMT2D and WDR5 defects are involved in Kabuki Syndrome characterized by multiple congenital abnormalities, from mild to severe developmental delay and intellectual disability. People suffering from this syndrome may also manifest seizures, hypotonia, strabism, hear infections, hearing loss and autism (Parisi et al., 2015). The very rare mutations in MANBA results in β-mannosidosis with a severe neurological disorder that can include mental retardation, cerebellar ataxia along with visual and hearing deficits (Sabourdy et al., 2009). CACNG3 is involved in Childhood Absence Epilepsy and is also associated with some cases of ASD (Danielsson et al., 2005) while mutations of KATNB1 cause complex cerebral malformations (Mishra-Gorur et al., 2014).

The remaining genes (among the 93) are mostly involved in epigenetics, cell cycle and cell adhesion and some of them are also implicated in tumor development as already reported by Crespi (2011) and Crawley et al. (2016).

The network-based analysis of genes from SFARI and other 5 previous studies resulted in the definition of a gene module that involves 561 ASD risk genes in significant functional relation. The module contains all the considered SFARI genes (strongly associated with ASD) and from 40% to 70% of genes from each of the other lists of ASD risk genes. Therefore, this module can be seen as a further screening of the genes proposed by such studies, which underlined those in significant functional relation from a network perspective.

More generally, the network-based scores that we calculated for every gene in the considered STRING network can be used to quantify the functional relation between any gene and ASD risk genes found in one or more previous studies.

Biological pathways enriched in genes in network proximity to ASD risk genes encompass several functions already proposed to be associated with ASD (see, for example, Pinto et al., 2010). Network-based analysis, through the prioritization of functionally related genes, enriched the number of significant pathways found by ORA in comparison to the analysis of original gene lists. Despite not all genes occurring in original lists underwent network-based analysis, the latter was not affected by a loss of information at pathway level.

The predicted genes in network proximity to ASD risk genes that have a central role in the PPI networks, but SRC, mapped in ASD risk loci. SFARI Gene database lists all the studies reporting CNV at the chromosome bands where predicted genes are localized (Table 4). In many reports, the CNV of interest was subsequently confirmed or validated by an independent method following its discovery. Additionally, from a functional point of view, most of the predicted genes are involved in epigenetics, cell cycle, growth-, proliferation- and differentiation-signaling and are often implicated in cancer development. This finding indicates pleotropic effects of some autism-associated genes on cancer risk and is supported by previous discussions that highlight a wide overlap in risk genes and pathways for cancer and autism (Crespi, 2011; Crawley et al., 2016). Advances in pharmacological therapies to ameliorate autism symptoms could be resulted from cancer drugs that target the same growth-signaling pathways (Crespi, 2011).

## Author contributions

EM collected the ASD data from the literature, setup and run the analyses; MB curated the physical mathematical modeling of network diffusion; MG and MM managed the high performance computing infrastructure; GC supported the physical mathematical modeling; LM coordinated the research; AM analyzed the ASD literature, interpreted the biological results, coordinated the research. All authors discussed the results, contributed to manuscript writing and revision.

### Conflict of interest statement

The authors declare that the research was conducted in the absence of any commercial or financial relationships that could be construed as a potential conflict of interest.
